# Observations on the Disorder of the General Health of Females Called Chlorosis, Showing the True Cause to Be Entirely Independent of Peculiarities of Sex

**Published:** 1839-10

**Authors:** 


ART. XV.—*Observations on the Disorder of the general Health of Females called Chlorosis, showing the true cause to be entirely independent of Peculiarities of Sex.* By SAMUEL FOX, Surgeon.—*London*, 1839. 8vo, pp. 130.

WE are somewhat disposed to publish a work to prove that a "chronic functional derangement of the liver" is the grand cause of all the indifferent or bad books which, as reviewers of medical literature, it is our fate to peruse. Our theory would be as follows. Individuals whose brains are, by temperament, inactive, are desirous of seeing their thoughts in actual print. They labour hard to think, and their cerebral powers are tried to the uttermost. Attention, memory, judgment, imagination—all diminish with the cerebral exhaustion. The direct nervous sympathy of the liver with the brain, or the indirect sympathy through the stomach, produces a languid action in that large and important viscus. The whole process of digestion is impaired, and consequently the blood which is required to keep up the energy of the brain is no longer of the requisite quality. The brain is thus, indirectly as well as directly, unfitted for its functions, and when the author attempts to put his thoughts into language, the faculties which had begun by diminishing are found to have utterly failed. He writes a bad book, and "chronic functional derangement of the liver" is the cause. Now the symptoms by which we judge of this derangement are headach, sleeplessness, exhaustion, loss of appetite, slow bowels, unhealthy stools, a feebleness and perhaps irregularity of circulation, &c.

This is a specimen of the kind of argumentation in the book before us; the object of which is, to prove, among other curiosities, that chronic functional derangement of the liver is chlorosis, and *vice versâ*. The absurdity of such reasoning may possibly strike the author in our theory, though it may escape him with regard to his own; for, unfortunately, the intellectual condition which permits an individual seriously to publish such an argument, unfits him for ascertaining its fallacy. In the present day, we can hardly think it worth while seriously to criticise such a book as the one before us; but, as the world is not yet quite freed of its "liver-doctors," and that Mr. Fox may not complain that we have not fairly treated him, we will take the trouble to expose his theory to his own view, apologising to our readers for thus occupying their valuable time:

"In describing," says Mr. Fox, "the symptoms of this chronic functional disorder of the liver, which I conceive to be the primary cause of these distressing ailments, (*i. e.* chlorosis), I must necessarily confine myself to those which are considered its leading, or more prominent features, while it would be an endless task to enumerate a multitude of sympathetic affections resulting from this disorder, which for the most part depend upon idiosyncracy; the greater number, however, of the symptoms, vary considerably as the disorder advances. At the commencement of this complaint there is generally a morbidly craving appetite, with but little thirst and fever; and, before there is much apparent loss of flesh, even whilst the appetite is verging on voracity, there is yet considerable debility, or rather an inaptitude for bodily exertion; though sometimes, for short periods, the patient is capable of using considerable exertion, owing, apparently, to a temporary mental stimulus. The disease insidiously gains ground, and the only alteration at first noticed by friends and relations, is the possession of less than usual animation, gradual loss of colour and complexion; sluggishness, (too often called laziness, for which the unfortunate patient is upbraided,) strong inclination for sleep, and great disinclination to rise in the morning; the patient throwing aside her usual amusements and occupations, becomes soon tired with slight exertions; frequently complaining of pains in her limbs. After a time, in many cases, there seems to be some degree of imbecility of intellect, as indicated by crying from slight causes, absence of mind, general inattention to surrounding objects, &c. Fetid breath, faintness, nightly perspirations, disturbed sleep, irritable stomach and nausea, are affections to be met with in almost every case of chronic functional derangement of the liver. The tongue is generally loaded, especially at its posterior part; sometimes, however, white, and sometimes particularly clean; the bowels are irregular, but when either loose or costive, the fecal evacuations are always unhealthy. The loss of strength and flesh now becomes real, and the body and legs are frequently swelled, whilst the latter, particularly about the ankles, are œdematous. By this time a vitiated appetite succeeds the former craving for food, and even when food is taken, it is not unfrequently ejected from the stomach. There is great depression of spirits, clammy perspiration about the face and breast, and especially about the nose and mouth; the complexion is now sallow, the eyes sunken, with dark areolæ formed around them, and the eye itself loses its wonted lustre. There is no discharge of the catamenia." (pp. 73-5.)

Before drawing Mr. Fox's serious attention to the foregoing extract from his volume, we may assure him that we have no particular theory respecting the nature of chlorosis to support, and that our object is simply to expose what appears to us to be obviously not merely incorrect, but absurd. We have little doubt that Mr. Fox, during his long practice, "extending over a period of between thirty and forty years," has found a large proportion of his patients very much satisfied to learn that their complaints are "bilious," and that he has likewise found it an effectual mode of escaping from difficulties of diagnosis, to ascribe symptoms connected with the nutritive, circulatory, nervous, or other systems, to sympathies with the liver. But we ask him, in considering with us the previous quotation, to endeavour to lay aside all preconceptions, to consider the subject, if possible, *de novo.* Among the early symptoms of this supposed functional liver affection is a "morbidly craving appetite," "but little thirst or pain," "debility, or rather inaptitude for bodily exertion;" "gradual loss of colour and healthy complexion," "sluggishness, strong inclination for sleep," "pains about the limbs," "imbecility of intellect," &c. On what sort of *evidence* does it rest that any one of the above symptoms arises from disordered hepatic function? We see distinctly a debilitated and depraved condition of the functions of the nervous and nutritive systems, but not a single symptom which we can satisfactorily associate with the liver. It may be true, as stated by Mr. Fox, that by some these are considered as the leading or most prominent features of chronic functional affection of the liver; but, by others, they are otherwise regarded, and we wish for the best demonstration of which the case will admit in deciding who are right. Sure we are that there is quite as much *proof* that the moon is made of green cheese, as that the above symptoms depend on chronic disorder of the liver. We consider it unnecessary to specify the other symptoms noticed by Mr. Fox, and which we have extracted. They may be variously explained; but we can find none among them, unless it be the characters of the fecal discharges, which cannot be quite as easily and rationally connected with other organs as with the liver. Having added a few more symptoms to those already mentioned, the author continues:

'This functional disorder of the biliary apparatus, I consider to be identical with the disordered state of health called chlorosis; and if we compare the symptoms of chlorosis, as described by Drs. Cullen, Mason Good, and others, with those above detailed, we shall find but little difficulty in assuming them as one and the same sort of symptoms." (p. 76.)

Correct pathology and common sense leave no other conclusion than that these notions of Mr. Fox are nonsense. We are sorry to pass so harsh a sentence on a work proceeding from a gentleman, no doubt, of great respectability, and who is, probably, even a successful practitioner; but Mr. Fox has evidently stepped beyond his line in turning author, and we counsel him to eschew all such follies for the future.

